# Prediction Model of Sound Signal in High-Speed Milling of Wood–Plastic Composites

**DOI:** 10.3390/ma15113838

**Published:** 2022-05-27

**Authors:** Weihua Wei, Yunyue Shang, You Peng, Rui Cong

**Affiliations:** College of Mechanical and Electronic Engineering, Nanjing Forestry University, Nanjing 210037, China; 17306396464@163.com (Y.S.); 18136883601@sina.cn (Y.P.); congrui240859@163.com (R.C.)

**Keywords:** wood–plastic composites, high-speed milling, sound signal, regression model, BP neural network, particle swarm optimization

## Abstract

The accuracy of the acoustic signal prediction model for wood–plastic composites milling has an important influence on the condition monitoring of the cutting process and the improvement of the machining environment. To establish a high-precision prediction model of sound signal in the high-speed milling of wood–plastic composites, high-speed milling experiments on self-developed wood–plastic composites were carried out with cemented carbide tools. A mathematical model of the relationship of the four milling parameters, including axial cutting depth, radial cutting depth, feed rate and cutting speed, and the sound signal of wood–plastic composites milling, was established by using the full-factor test method. The experimental data obtained by the orthogonal test method were used as the test samples in the mathematical model. Test results show that the prediction accuracy of the mathematical model of the sound signal in the milling of wood–plastic composites exceeds 95.4%. To further improve the prediction accuracy of the sound signal in the milling of wood–plastic composites, a prediction model was established using back propagation (BP) neural network. Then, the particle swarm optimization (PSO) algorithm was used to optimize the BP neural network, obtaining the PSO–BP neural network prediction model. The test results show that the prediction accuracy of the PSO–BP prediction model for the sound signal in the high-speed milling of wood–plastic composites exceeds 97.5%. The PSO–BP model has a better global approximation ability and higher prediction accuracy than the BP model. The research results can provide a reference basis for sound signal prediction in the high-speed milling of wood–plastic composites.

## 1. Introduction

Given the shortage of natural wood resources, some artificial products have gradually appeared in the market for use as a natural wood replacement. Wood–plastic composites (WPCs) are composite materials that combine wood-based materials and polymers, which have the following advantages: anti-corrosion, moisture resistance, insect resistance, strong fire resistance, high dimensional stability, crack resistance and low cost [1,2]. Therefore, WPCs are used to replace wood in many industries, such as in the daily necessities and civil engineering fields [3,4]. As an advanced manufacturing technology, high-speed cutting technology can improve production efficiency, machining accuracy and surface quality, and reduce cutting force and production cost, and has been widely used in the cutting of wood materials [5]. Given the anisotropy and heterogeneity of WPCs, obvious vibration and noise are easily produced during high-speed milling, which leads to the deterioration of the machining environment and even makes the machining accuracy and surface quality of parts difficult to guarantee [6]. Therefore, establishing a high-precision prediction model of noise in the high-speed milling of WPCs and reducing the noise in high-speed machining by selecting appropriate cutting parameters are of great significance. Sampath et al. [7,8] used a finite element model to predict the sound pressure generated during the high-speed end milling of aluminum alloy and found that cutting parameters and tool geometry parameters have important effects on noise. Karakurt et al. [9] used multiple regression analyses to establish a noise model and found that cutting speed has the greatest influence on noise level among the cutting parameters. Ji et al. [10] established a prediction model of aerodynamic noise on the basis of the Navier–Stokes equation and studied the influence of tool geometry parameters on the performance of milling cutters. Their results show that the number of teeth of milling cutters has a significant effect on noise. Darmawan and Tanaka [11] found that the sound pressure level (*SPL*) when cutting wood panels increases gradually with tool wear and multiple sources of noise exist in the background environment during actual machining. During high-speed cutting, due to the simultaneous influence of tools, workpiece clamping and other factors, a complex non-linear relationship exists between independent variables and the response, which cannot be described accurately by the statistical regression method alone. Therefore, the prediction model based on neural networks has been widely used [12,13]. The BP neural network is a multi-layer feed-forward neural network trained by the error back propagation algorithm, which is suitable for prediction and classification [14,15,16]. Paul and Varadarajan [17] established regression and BP neural network models through AISI4340 steel turning experiments and found that the prediction accuracy of the model based on the artificial neural network is better than that of the regression model. Although the neural network has unique advantages in dealing with multiple factors and nonlinearity, it is prone to problems, such as slow convergence and ease of falling into a local optimal solution [18,19]. There are many kinds of optimization methods, such as Particle Swarm Optimization (PSO), the Dragonfly Algorithm (DA), the Grey Wolf Optimization Algorithm (WOA), Elephant Herding Optimization (EHO), Monarch Butterfly Optimization (MBO), the Moth Search Algorithm and Harris Hawks Optimization (HHO) [20]. The particle swarm optimization (PSO) algorithm has the advantages of fast convergence, few parameters and easy implementation. It can optimize the weight and structure of the neural network, avoid falling into a local optimal solution, and expand the search range and improve the efficiency of calculation [21,22]. In addition, for the selection of time domain and frequency domain, Adpa et al. [23] considered that it was reasonable to select only the time domain response of sound signal over frequency and time–frequency, after consulting a large number of studies.

In summary, the studies above mainly focused on wood and metal materials, whereas no research on the sound signal prediction model for the high-speed milling of WPCs has been reported. Given the characteristics of anisotropy and heterogeneity, the evolution mechanism of the sound signal generated by WPCs in high-speed milling is complex. To establish a high-precision prediction model of the high-speed milling sound signal of WPCs, this study investigated the relationship between the milling parameters and the high-speed milling sound signal of WPCs through high-speed milling experiments. A full-factor test method was designed to establish a mathematical regression model of the sound signal in the high-speed milling of WPCs; an orthogonal experiment scheme was designed to test the prediction accuracy of the regression model with the obtained experimental data. The BP neural network was used to establish the prediction model of the sound signal in the high-speed milling of WPCs and PSO was used to optimize the BP neural network to establish a highly precise prediction model. The research results are of great significance for reducing noise, optimizing the machining environment and ensuring machining quality in the high-speed milling of WPCs.

## 2. Experimental Scheme and Design

### 2.1. Experimental Conditions

**Figure 1:**
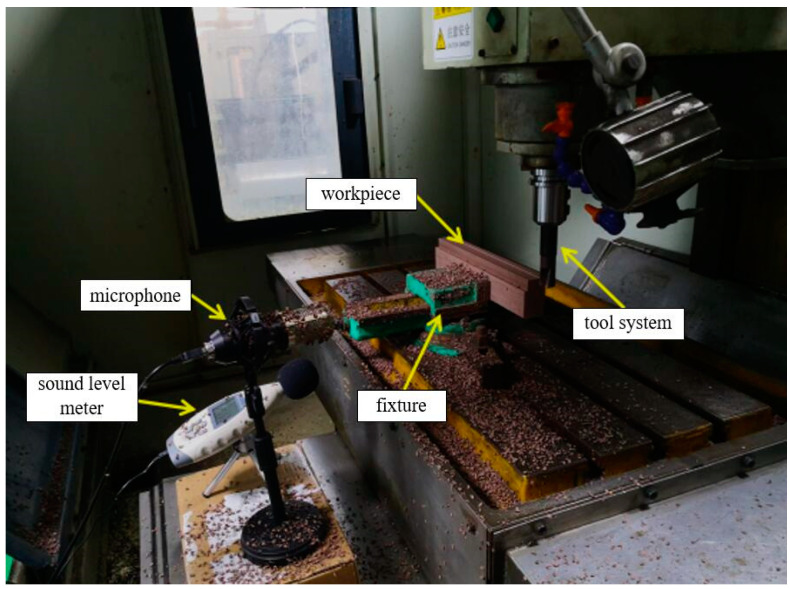
Milling sound signal acquisition system.

The high-speed milling experiment was carried out at an XH714 machining center. The type of side-fixed shank was BT40-SLN25 and the milling cutter rod was BAP40r. A carbide blade was mounted on the milling cutter rod and the model of the blade was APMT1604PDER-K22510P. The workpiece size was 320 mm × 80 mm × 40 mm, which was produced by Nanjing Dayuan Plastic Wood New Material Co., Ltd. (Nanjing, China). The composition and main performance parameters of the WPCs are shown in Table 1 and Table 2, respectively. A microphone was used to record the milling sound signal and an Ar844 Hima sound level meter was used to measure the milling sound pressure level. The maximum sound frequency that a human can hear is about 20,000 Hz. Nyquist’s sampling theorem requires that the sampling frequency must be higher than 2 times the highest frequency of the input signal in order to restore the original signal without distortion or retaining the information completely. Therefore, the sampling frequency was 44,100 Hz [24]. The milling method adopted was reverse milling and the milling sound signal acquisition system is shown in Figure 1.

### 2.2. Experimental Design

**Figure 2:**
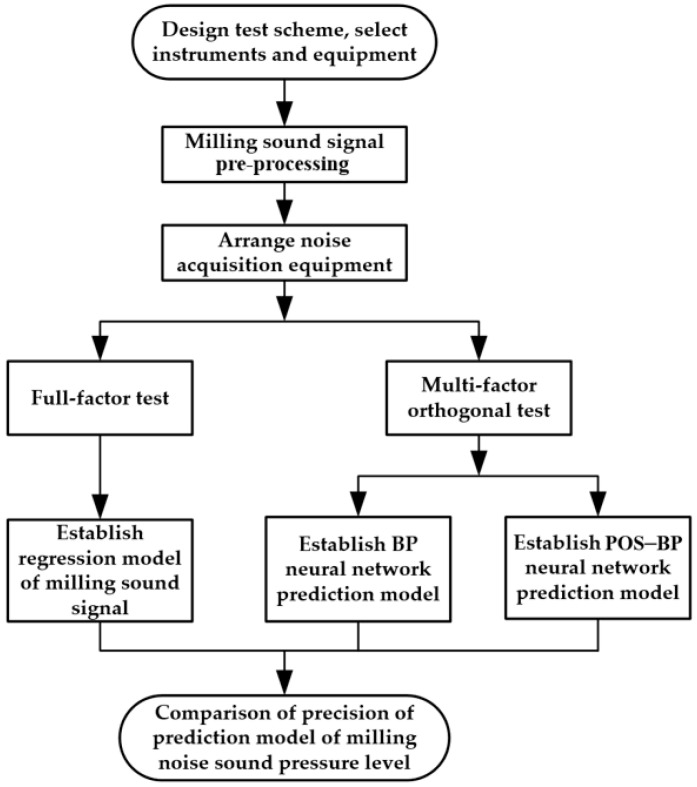
The pictorial flowchart of the overall framework.

The full-factor test and orthogonal test were used in this study [25]. The test schemes are shown in Table 3 and Table 4, respectively. In the experiment, axial cutting depth *a_p_*, radial cutting depth *a_e_*, feed rate *f* and cutting speed *v* were used as input variables and milling sound pressure level *SPL* was used as the output variable. The maximum sound pressure level of the idle noise of the spindle was collected before each group of tests as the background noise sound pressure level and the maximum sound pressure level within the milling time of each group was collected as the milling process sound pressure level. To reduce the contingency of the validation model’s accuracy, the data of the full-factor test scheme were selected as the training data of the milling sound signal model and the data of the orthogonal test scheme were used as test samples to verify the prediction accuracy of the model. The pictorial flowchart of the overall framework is shown in Figure 2 [26].

## 3. Milling Sound Signal Pre-Processing

**Figure 3:**
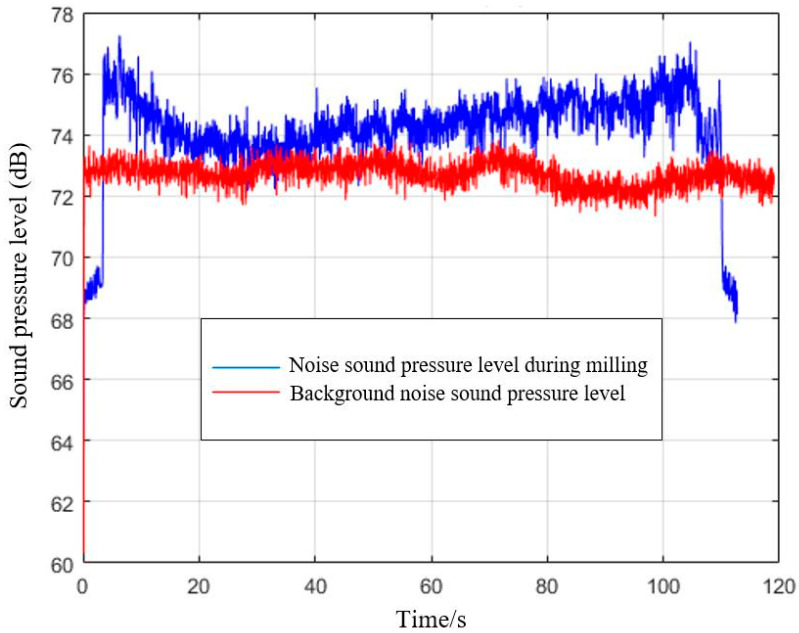
Sound pressure level curve of milling process noise and background noise.

The curves of the milling process and background noises are shown in Figure 3. The figure shows that the sound pressure level of the background noise is near that of the milling process, which overlap each other in some periods. Background noise affects the milling noise, making the removal of the interference of background noise using the decibel subtraction method necessary. In this test, the milling process noise was taken as the total noise and the spindle idle noise was taken as the main background noise. The milling sound signal was processed to eliminate the background noise. The milling sound pressure level was calculated by Formula (1).

(1) $$SPL=10\mathrm{lg}(10^{\frac{SPL_{1}}{10}}-10^{\frac{SPL_{2}}{10}}),$$

where:

*SPL*—Milling sound pressure level, Db;

*SPL*_1_—Total noise pressure level during milling, dB;

*SPL*_2_—Background noise sound pressure level, dB.

After removing the background noise sound pressure level, the milling sound pressure level test data obtained by the full-factor test scheme are shown in Table 5, and the milling sound pressure level test data obtained by the multi-factor orthogonal test scheme are shown in Table 6.

## 4. Regression Model and Verification of Milling Sound Signal in Full-Factor Test

The Design-Expert software was used to analyze the test data in Table 5 and determine the significant influence level on the milling sound pressure level. The items with *p* ≤ 0.05 were substituted into the model and the items with *p* > 0.05 were eliminated. The milling sound pressure level was fitted by multiple linear regression, and its basic form is as follows:

(2) $$SPL=A_{0}+\sum_{i=1}^{n}A_{i}x_{i}+\sum_{i=1}^{n}A_{ij}x_{i}x_{j},$$

where *SPL* is the milling sound pressure level, *A*_0_ is a constant term, *A_i_* represents the coefficients of the linear terms and *A_ij_* represents the coefficients of the interactive terms.

The unknown coefficients were calculated according to the test data in Table 5, and the regression model of the milling sound pressure level was obtained as follows:

(3) $$SPL=70.4+0.67a_{p}-0.27a_{e}+0.04v+0.58f+0.11a_{p}a_{e}+1.05a_{e}f,$$

To reduce the contingency of the prediction results of the model and compare the results with the PSO—PB and PB models, the 18th to 25th groups of test data in Table 6 were taken to verify the regression model. The verification results are shown in Table 7. The error between the predicted and real values of the milling sound pressure level is within 4.6% and the total mean value of the prediction error is 2.29%.

## 5. Prediction Model of Milling Sound Signal Based on BP Neural Network Optimized by PSO

### 5.1. Construction of BP Neural Network Prediction Model

**Figure 4:**
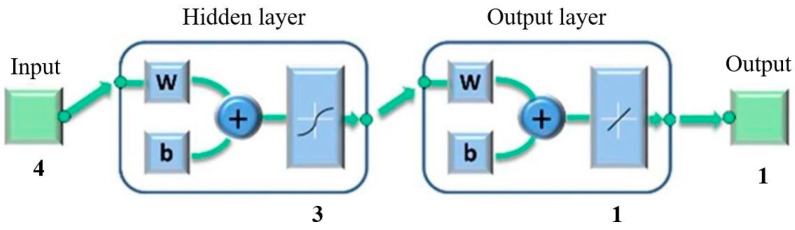
BPNN model topological structure.

This study adopted a three-layer neural network, including input and output layers and a hidden layer [27]. The topological structure of the BPNN model in this work is shown in Figure 4.

**Figure 5:**
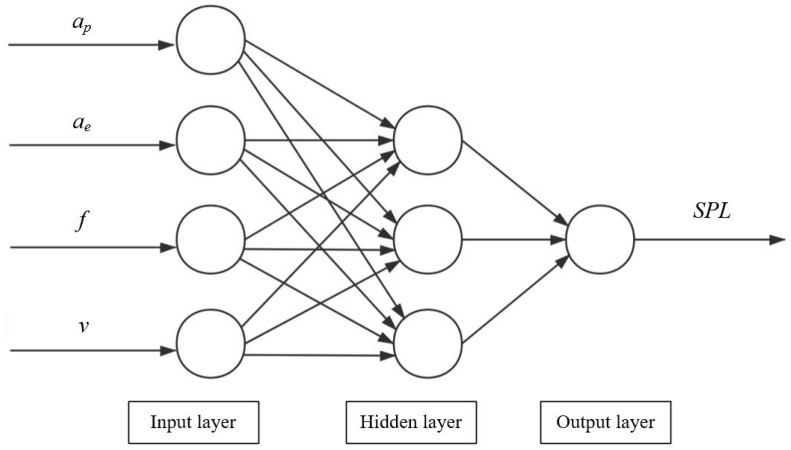
Network structure.

Taking *a_p_*, *a_e_*, *f* and *v* as input variables and *SPL* as the output variable, the schema of the network structure is shown in Figure 5. The number of neurons in the input layer of the neural network is 4 and that in the output layer is 1. In this study, the empirical Formula (4) was used to find the optimal number of neurons [28] and the number of neurons in the hidden layer was found to be between 2 and 12. The software used in training was Matlab. After many tests, the prediction effect of the model was determined to be the best when the number of hidden layer neurons is 3. The training parameters are shown in Table 8.

(4) $$\begin{array}{l} H=\sqrt{m+n}+\alpha \\ H=\log_{2}m\\ H=\sqrt{m\left(n+2\right)}+1 \end{array}$$

where *H* is the number of neurons in the hidden layer, *m* is the number of neurons in the input layer, *n* is the number of neurons in the output layer, *α* is a constant and *α*∈[1, 10].

### 5.2. Optimization of BP Neural Network by PSO

PSO constructs a new intelligent optimization algorithm by simulating the cooperative optimization ability of swarm organisms. PSO is initialized as a group of random particles, which have only two properties: velocity and position. In each iteration, particles update themselves by tracking two “extreme values” (*pbest*, *gbest*). After finding the two optimal values, the particle updates its velocity and position through Formulas (5) and (6), respectively.

(5) $$v_{i}=v_{i}+c_{1}\times rand()\times (pbest_{i}-x_{i})+c_{2}\times rand()\times (gbest_{i}-x_{i}),$$

(6) $$x_{i}=x_{i}+v_{i},$$

where *v_i_* is the velocity of the particle, *pbest* is the extreme value of the particle itself, *gbest* is the global extreme value, *rand* () is a random number between (0,1), *x_i_* is the position of the particle, and *c*_1_ and *c*_2_ are acceleration constants. As an optimization algorithm, the PSO algorithm can search and obtain the optimal initial weight and threshold value of the BP neural network. After a considerable amount of experimental training, the optimal parameter values are as shown in Table 9.

### 5.3. Network Training and Verification

In the process of constructing a neural network, datasets need to be divided. There are various ratios between the training set and the test set, such as 80:20, 70:30 and 60:40 [29]. When the amount of data is small and there is no verification set, the ratio between the training set and the test set is usually 70:30. After determining the parameters of the PSO–BP model, the results in the multi-factor orthogonal test table were selected as the neural network training data, with 70% and 30% of the test results as the respective training and test samples. The network training samples are data No. 1–17 in Table 6. The network test samples are data No. 18–25 in Table 6. It is generally necessary to disarrange the order of original sample data to test it. The trained model was used to classify datasets [30].

The data of the test sample were substituted into the constructed neural network to verify the neural network model. The results are shown in Table 10. The total mean values of the prediction error of the BP and PSO–BP neural network models are 1.31% and 0.94%, respectively. Spss was used to analyze the BP-predicted value, the PSO–BP-predicted value and the true value in Table 10. The comparison results of the two neural network models are shown in Table 11. The prediction value of the BP neural network optimized by PSO was nearer the real value and the determination coefficient R^2^ increased from 0.83 to 0.93, exhibiting a better global approximation ability than the BP neural network. According to Table 11, we can conclude that the error of the PSO–BP neural network model prediction results was further reduced and the prediction accuracy increased.

## 6. Conclusions

(1) The mathematical regression model (Equation (3)) of the sound signal in the WPC milling process was established by using the full-factor test method. The test results show that the prediction error of the regression model is less than 4.6%, indicating good prediction ability.
(2) The PSO algorithm was used to optimize the BP neural network prediction model of the sound signal in the WPC milling process, obtaining the PSO–BP prediction model. Compared with the BP neural network, the determination coefficient (R^2^) of the PSO–BP neural network prediction model increased from 0.83 to 0.93 and the prediction accuracy improved significantly. Therefore, during the high-speed milling of WPCs, the PSO–BP neural network prediction model has a better global approximation ability than the BP neural network model.
(3) In the sound signal prediction model for the WPC milling process, the total mean values of the prediction error of the PSO–BP model decreased by 59% and 28%, respectively, compared with the regression and traditional BP neural network models. Therefore, the PSO–BP neural network model can effectively improve the prediction accuracy of WPCs’ milling sound signal and provide a highly precise mathematical model for predicting the variation law of the sound signal in the high-speed milling of WPCs.

## Figures and Tables

**Table 1:** Composition of WPCs.

Material Composition	Wood Flour	Calcium Carbonate	PE Used Material	Phase Solvent	Lubricant
Proportion	54.70%	13.70%	27.35%	2.74%	1.51%

**Table 2:** Main performance parameters of WPCs.

Density (g/cm^3^)	Flexural Modulus (MPa)	Shore Hardness (HD)
1.19	28	58

**Table 3:** Full-factor test level.

Name	Unit	Low Level	High Level
*a_p_*	mm	3	5
*a_e_*	mm	6	10
*v*	m/min	250	350
*f*	mm/r	0.1	0.5

**Table 4:** Orthogonal test level.

Horizontal Number	*a_p_*	*a_e_*	*v*	*f*
	(mm)	(mm)	(m/min)	(mm/r)
1	1	2	150	0.1
2	2	4	200	0.2
3	3	6	250	0.3
4	4	8	300	0.4
5	5	10	350	0.5

**Table 5:** Full-factor test data.

Std	Test Number	*a_p_*	*a_e_*	*v*	*f*	*SPL*
		(mm)	(mm)	(m/min)	(mm/r)	dB
8	1	5	10	350	0.1	77.7
4	2	5	10	250	0.1	74.1
13	3	3	6	350	0.5	80.2
12	4	5	10	250	0.5	90.2
16	5	5	10	350	0.5	72.8
9	6	3	6	250	0.5	82.2
15	7	3	10	350	0.3	80.8
10	8	5	6	250	0.3	83.3
5	9	3	6	350	0.1	81.9
17	10	4	8	300	0.1	83.5
19	11	4	8	300	0.5	82.3
3	12	3	10	250	0.5	93.6
1	13	3	6	250	0.1	77.1
6	14	5	6	350	0.1	68.3
14	15	5	6	350	0.3	82.5
7	16	3	10	350	0.5	85.5
11	17	3	10	250	0.1	89.1
18	18	4	8	300	0.5	79.4
2	19	5	6	250	0.1	68.5

**Table 6:** Multi-factor orthogonal test data.

Test Number	*a_p_*	*a_e_*	*v*	*f*	*SPL*
	(mm)	(mm)	(m/min)	(mm/r)	dB
1	1	2	150	0.1	67.2
2	1	4	200	0.2	69.4
3	1	6	250	0.3	73.9
4	1	8	300	0.4	84.9
5	1	10	350	0.5	82.4
6	2	2	200	0.3	80.5
7	2	4	250	0.4	81.3
8	2	6	300	0.5	81.5
9	2	8	350	0.1	77.7
10	2	10	150	0.2	79.7
11	3	2	250	0.5	83.8
12	3	4	300	0.1	78.3
13	3	6	350	0.2	84.1
14	3	8	150	0.3	82.6
15	3	10	200	0.4	87.7
16	4	2	300	0.2	91.8
17	4	4	350	0.3	75.6
18	4	6	150	0.4	85.4
19	4	8	200	0.5	89.2
20	4	10	250	0.1	79.9
21	5	2	350	0.4	84.1
22	5	4	150	0.5	85.6
23	5	6	200	0.1	80.4
24	5	8	250	0.2	84.8
25	5	10	300	0.3	88.1

**Table 7:** Error table between prediction results and real values.

Number	*a_p_*	*a_e_*	*v*	*f*	Prediction Results	Real Values	Error Percentage
	(mm)	(mm)	(m/min)	(mm/r)	dB	dB	%
18	4	6	150	0.4	81.7	85.4	4.6
19	4	8	200	0.5	86.4	89.2	3.3
20	4	10	250	0.1	81.5	79.9	1.9
21	5	2	350	0.4	85.7	84.1	1.8
22	5	4	150	0.5	83.4	85.6	2.7
23	5	6	200	0.1	81.2	80.4	1.1
24	5	8	250	0.2	85.3	84.8	0.6
25	5	10	300	0.3	90.0	88.0	2.3

**Table 8:** BP neural network training parameters.

*m*	*n*	Number of Hidden Layers	*H*	Transfer Function		Training Function
				Hidden Layer	Output Layer	
4	1	1	3	*logsig*	*purelin*	*trainlm*

**Table 9:** PSO parameters.

Population Size	Evolution Algebra	*c*_1_	*c*_2_
20	40	1.49618	1.49618

**Table 10:** Prediction accuracy verification results.

Number	*a_p_*	*a_e_*	*v*	*f*	BP Prediction Values	PSO–BP Prediction Values	Real Values	BP Error Percentage	PSO–BP Error Percentage
	(mm)	(mm)	(m/min)	(mm/r)	(dB)	(dB)	(dB)	(%)	(%)
18	4	6	150	0.4	87.1	86.7	85.4	1.9	1.50
19	4	8	200	0.5	87.6	89.5	89.2	1.8	0.40
20	4	10	250	0.1	81.3	78.2	79.9	1.7	2.10
21	5	2	350	0.4	83.4	84.3	84.1	0.9	0.13
22	5	4	150	0.5	87.5	86.1	85.6	2.2	0.60
23	5	6	200	0.1	80.8	80.8	80.4	0.5	0.51
24	5	8	250	0.2	84.7	83.1	84.8	0.2	2.00
25	5	10	300	0.3	86.8	87.8	88.0	1.3	0.30
Mean error percentage								1.3	0.94

**Table 11:** Comparison of two neural network models.

Model	R	R Square	Adjusted R Square	Std. Error of the Estimate
BP	0.91	0.83	0.80	1.24
PSO–BP	0.96	0.93	0.92	1.08

## Data Availability

Not applicable.
